# Cortical networks with multiple interneuron types generate oscillatory patterns during predictive coding

**DOI:** 10.1371/journal.pcbi.1013469

**Published:** 2025-09-10

**Authors:** Kwangjun Lee, Cyriel M. A. Pennartz, Jorge F. Mejias

**Affiliations:** 1 Cognitive and Systems Neuroscience Group, Swammerdam Institute for Life Sciences, University of Amsterdam, Amsterdam, the Netherlands; 2 Research Priority Area Amsterdam Brain and Cognition, University of Amsterdam, Amsterdam, the Netherlands; Stem Cell and Brain Research Institute: Cellule Souche et Cerveau, FRANCE

## Abstract

Predictive coding (PC) proposes that our brains work as an inference machine, generating an internal model of the world and minimizing predictions errors (i.e., differences between external sensory evidence and internal prediction signals). Theoretical models of PC often rely on high-level approaches, and therefore implementations detailing which neurons or pathways are used to compute prediction errors or adapt the internal representations, as well as their level of agreement with biological circuitry, are currently missing. Here we propose a computational model of PC, which integrates a neuroanatomically informed hierarchy of two cortical areas with a simplified laminar organization and cell-type-specific connectivity between excitatory, PV, SST and VIP cells. Our model efficiently performs PC, even in the presence of external and internal noise, by forming latent representations of naturalistic visual input (MNIST, fashion-MNIST and grayscale CIFAR-10) via Hebbian learning and using them to predict sensory input by minimizing prediction errors. The model assumes that both positive and negative prediction errors are computed by stereotypical excitatory-PV-SST-VIP circuits with the same structure but different incoming input. During sensory inference, neural oscillatory activity emerges in the system due to interactions between representation and prediction error microcircuits, with optogenetics-inspired inactivation protocols revealing a differentiated role of PV, SST and VIP cell types in such dynamics. Finally, our model shows anomalous responses to deviant stimuli within series of same-image presentations, in agreement with experimental results on mismatch negativity and oddball paradigms. We argue that our model constitutes an important step to better understand the circuits mediating PC in real cortical networks.

## Author summary

Predictive coding (PC) suggests that the brain constantly generates expectations about the world and updates these expectations based on incoming sensory input. While being a prominent theory, we still lack detailed models that show how specific neurons and brain circuits might carry out these processes. Here, we present a computational model which addresses this gap by including biologically plausible brain circuitry with specific types of neurons (excitatory, PV, SST, and VIP cells) and their connections. It efficiently learns to form internal representations of visual information and uses them to predict sensory input, adjusting its predictions when errors occur. We found that particular types of neurons play different roles in these processes, and that neural oscillations emerge during the training and inference processes. Our model also replicates neural patterns observed in experiments where unexpected stimuli appear. By integrating anatomical and functional details, our work brings us closer to understanding how the brain uses predictive coding at the circuit level.

## Introduction

With only indirect access to the external world via information from the senses, the brain must infer the properties of objects based on the neural responses they elicit. This process of inference, aimed at determining how the external world (distal stimulus) causes sensory signals (proximal stimulus), involves extracting the external world’s underlying structure (i.e., ‘causes’) and representing it with abstract neural signals (‘latent representations’). The inferential nature of the perceptual experience opens the door to ill-posed problems when dealing with noisy and ambiguous sensory signals. A potential solution to the problem of maintaining a seamless perceptual experience is provided by predictive coding (PC), which proposes that the brain constantly generates and updates an internal model of the world to predict incoming sensory inputs [1–3].

Central to PC is the idea that cortical circuits minimize the discrepancy between the actual and predicted sensory inputs (i.e., the prediction error). This working principle has been explored in many experimental and computational studies on PC [4–21]. Despite early efforts to identify canonical cortical circuits for predictive coding [6], implementations have often followed functional rather than neurobiological guidelines. Thus, it remains unclear how PC may be carried out by cortical networks. Recently, progress has been made in the identification of different inhibitory interneuron types in neocortex, such as Parvalbumin- (PV), somatostatin- (SST), and/or vasoactive intestinal peptide (VIP) expressing cells, as key players in microcircuits computing prediction errors [4,13,22–25]. Several studies suggest that computations might happen independently for positive prediction errors (i.e., when sensory signals exceed the internal prediction) and negative prediction errors (i.e., when internal prediction exceeds sensory signals) via different circuits [12,14,23,25–27], providing insight in the underlying PC circuitry beyond classical frameworks. However, we are still missing a complete mapping of PC circuitry using neurobiologically plausible principles, i.e., not only the local circuitry for computing prediction errors comprising excitatory (E), PV, SST, and VIP cells but also the circuitry for embedding those local configurations in realistic hierarchical cortical networks and adapting the brain’s internal world models as a result of such predictions.

In the present study, the primary objective is to propose and analyze a mechanistically plausible circuit implementation for core predictive coding principles. The focus is on demonstrating how fundamental PC computations, such as prediction error generation and hierarchical inference, can emerge naturally from the dynamic interactions within a neurobiologically grounded cortical microcircuit, rather than aiming for the precise replication of a specific experimental dataset. To achieve this, we integrate neuroanatomically informed projections patterns [28,29] with a simplified framework for the laminar organization and cellular diversity of neural circuits in sensory cortex to provide a fully-fledged PC model for visual perception. Our model is constituted by a hierarchy of two interconnected cortical areas in which positive and negative prediction errors are computed in two coexisting circuits located in superficial cortical layers of each area. Both circuits are described by the same minimal connectivity pattern (i.e., a closed E-PV loop and a VIP → SST → E disinhibitory circuit [30], but differ in the particular cell types targeted by incoming synapses from other areas. Upon presenting visual input to our model and subjecting it to Hebbian plasticity, we demonstrate that this architecture forms latent representations of naturalistic images (MNIST, fashion-MNIST and grayscale CIFAR-10) and reconstructs input images while minimizing prediction errors during sensory inference, and that its performance is robust against both external and internal sources of noise. The inspection of the resulting neural dynamics reveals the emergence of neural oscillations during sensory inference, with a frequency which may range from alpha (10 Hz) to gamma (>30 Hz), depending on hyper-parameter settings. These oscillations are of the stable node (or ‘damped oscillator’) type and emerge due to the recurrent interaction between representation and error neurons. The emergence of oscillations reflects the neurobiologically realism of the model and agrees with recent proposals linking alpha-band oscillations to top-down predictions [31]. When presented with a series of the same stimuli interrupted by occasional deviant stimuli, our model also displays anomalous responses to deviants as reported for standard oddball paradigms [32–40], with the strength of the anomalous response depending on the position of the deviant in the series. This demonstrates how such effects could potentially arise from the proposed circuit dynamics. Finally, selective inactivation of each cell type in the network (mimicking optogenetic experiments) provides predictions regarding their distinct functional roles in PC: while PV provides a blanket of inhibition dampening network activity, SST and VIP serve to control the amplitude of neural oscillations and the number of cycles between representation and error neurons in opposing directions. Taking all these results together, the present model provides a neurobiologically plausible architecture for PC, highlighting the role of different interneuron types and the resulting oscillatory dynamics.

## Results

### Cortical circuits for predictive coding

**Fig 1:**
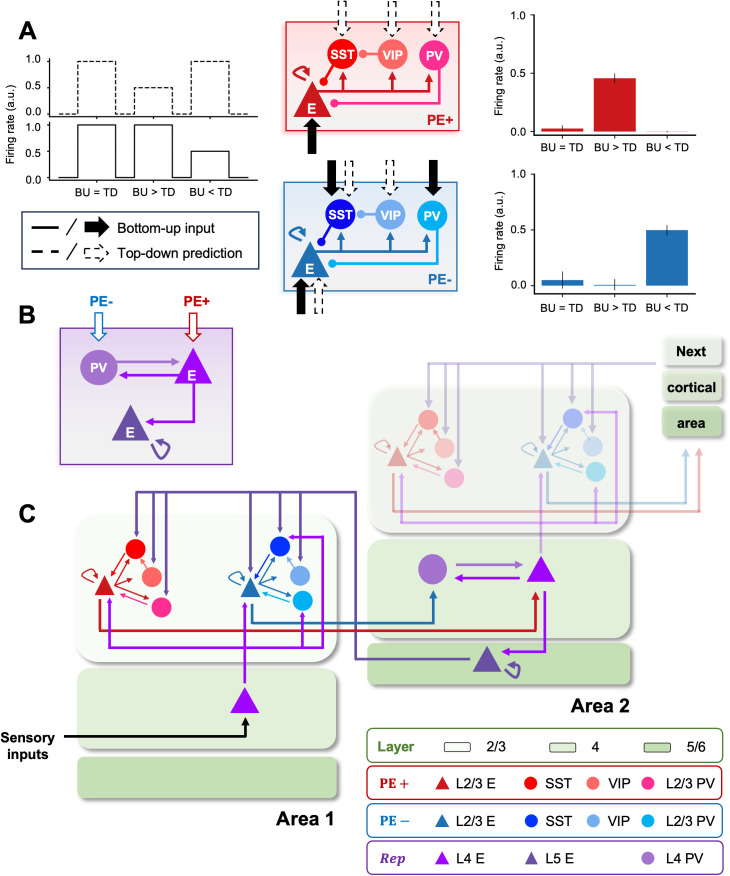
Cortical column model of predictive coding. The cortical column model of predictive coding consists of prediction error and representation microcircuits. (A) The connectivity patterns among four cortical neuron types (E, PV, SST, and VIP) within two types of prediction error microcircuits as well as synaptic inputs each of those neurons receive were determined via a combinatorial search. The canonical microcircuits (middle panel) have the same within-circuit connectivity but compute either positive (BU > TD) or negative (TD < BU) prediction errors (right panel; shown in red and blue, respectively), depending on synaptic input patterns. (B) The representation microcircuit receives the two types of prediction errors using excitatory and PV cells. The recurrent connection between them enables the integration of the two errors to update the latent representation of sensory inputs. (C) The laminar positioning of prediction error and representation microcircuits mediating predictive coding of sensory inputs. Prediction error microcircuits are situated in layer 2/3, whereas the representation microcircuit is in layer 4/5. A higher area (e.g., Area 2) receives positive and negative prediction errors from layer 2/3 of a lower area (e.g., Area 1) to update internal representations and make predictions of incoming sensory inputs in a lower area via feedback projections from layer 5 excitatory cells (whose role is to faithfully relay this top-down signal). While the model restricts the cortical hierarchy to two levels, it can be expanded to multiple columns as depicted in the upper right opaque image in Area 2 and the next cortical region.

To propose a biologically plausible framework for predictive coding, we built a network model with circuit level detail to perform PC by incorporating canonical cortical circuits that consist of excitatory (E) neurons and three types of inhibitory interneuron: PV, SST, and/or VIP cells [4,41] - instead of the arbitrary computational units of prediction error and representation units from previous work [2]. Prediction error microcircuits computed the discrepancy between bottom-up sensory inputs and top-down predictions. These prediction errors (PEs) are positive when bottom-up sensory inputs are greater than top-down predictions and negative if vice versa (BU > TD versus BU < TD, respectively; Fig 1A). To reflect the non-negative nature of spike signalling in the brain, PEs were further divided into positive and negative subgroups. Empirical evidence supports such grouping of neurons [25–27]. Our previous work [14] also showed that implementing separate neuronal populations for positive and negative prediction errors indeed can implement PC in spiking neural networks.

To determine the connectivity and synaptic input patterns of prediction error microcircuits, we ran a combinatorial search to identify those circuits that allow exclusive encoding of positive or negative prediction errors. Our results showed that, despite sharing the same connectivity pattern among the four cell types within their circuits, the differences in sensory input patterns ensured that excitatory neurons from the two subgroups increased firing rates to exclusively encode either type of error (Fig 1A). Meanwhile, the representation microcircuit was hard-wired to continuously generate and update latent representations of bottom-up inputs (Fig 1B). The recurrent connectivity between layer 4 excitatory and PV cells integrated the two types of errors. Layer 5 excitatory cells sent top-down projections to lower areas, fulfilling the role of faithfully relaying the predictions to lower areas. This circuit is able to form internal representations (in the form of spatial activity patterns) of sensory stimuli and generate internal predictions to match them.

A key contribution of PV, SST and VIP interneurons is that they play an important role in generating the positive and negative prediction error responses (the rightmost panels of Fig 1A). For example, for negative prediction error circuits, excitatory activity gets strongly suppressed by PV and SST activity when bottom-up input is stronger than top-down. However, if top-down input is stronger, VIP activity suppressed SST cells and the excitatory drive may overcome PV activity, resulting in a strong activity reflecting a negative prediction error. For the case of positive prediction error circuits, strong bottom-up input triggers a significant positive prediction error response (i.e., bottom-up input larger than top-down), while for strong top-down input, the activation of PV cells and the lack of any SST effects (due to VIP suppression) leads to an inhibition of excitatory neurons. In practice, the level of bottom-up and top-down signals will determine whether positive or negative prediction errors are generated, as well as their strength.

### Laminar placement of cortical circuits

Our model consisted of two adjacent cortical areas positioned along the visual cortical hierarchy (Fig 1C). Each area in the cortical hierarchy corresponds to a cortical column that consists of supra-granular layers (i.e., layer 2/3) harboring PE microcircuits, and granular and infra-granular layers (i.e., layer 4/5) containing representation microcircuits. The laminar position and projection patterns within and between cortical columns followed the anatomy of the neocortex [6,28].

In the first, lower cortical area (e.g., V1), thalamic sensory inputs are fed to layer 4 excitatory neurons (black triangle; Fig 1C) and relayed to layer 2/3. Predictions regarding these bottom-up inputs are transmitted from layer 5 of the second, higher cortical area (e.g., V2; purple excitatory cell). The disparities between the actual and predicted sensory inputs (PE+ and PE-) are computed in layer 2/3 of the first area and conveyed back to layer 4 of the second column as prediction error signals (red lines) to refine latent representations of the bottom-up inputs. Note that scaling beyond the second cortical column only requires stacking up the PE feedback loop (see the upper right part of Fig 1C with a lower opacity). However, we limited the cortical hierarchy to two areas to emphasize the laminar architecture and cortical circuitry with interneurons that mediate predictive coding.

### Reconstruction of sensory inputs via predictive coding

**Fig 2:**
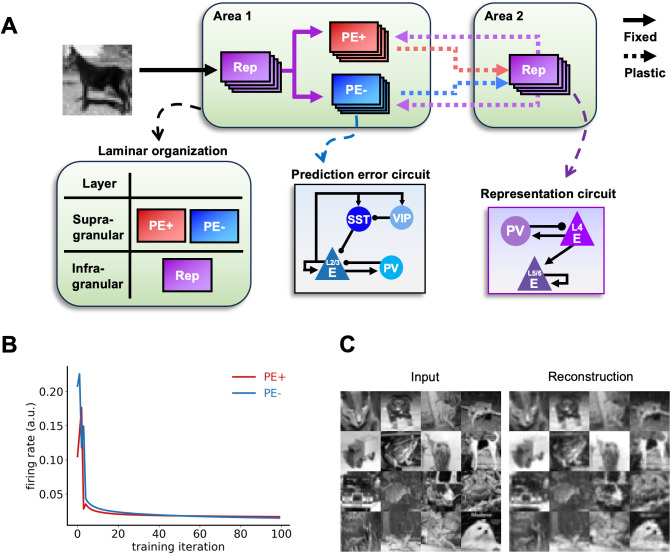
Perceptual inference and learning of naturalistic images. (A) A summary schematic of the cortical column model of predictive coding, with details on the laminar organization, prediction error circuits and representation circuits. (B) Prediction error minimization across training iterations indicates that representation learning is taking place. Both positive and negative prediction errors decrease monotonically (red and blue lines, respectively). Note that the lines indicate excitatory neuron activity from each prediction error microcircuit. (C) The model reconstructs images unseen during training, despite having been presented with only a small subset of the entire image dataset (containing 4.3% of total training images). Our model, embedded with biological constraints like laminar structure, anatomical projections and neuronal diversity, was therefore able to extract the underlying statistics from naturalistic images and learned to generate latent representations.

To test whether our model can perform perceptual inference and learning of sensory inputs in the present architecture, which includes laminar organization, cortical circuits with interneurons, and anatomical projection patterns, we trained it with a well-known image dataset (CIFAR-10 dataset; Fig 2A) and examined its performance in prediction error minimization (Fig 2B) and image reconstruction (Fig 2C).

Except for the initial fluctuations, the monotonically decreasing prediction errors across training iterations (Fig 2B) as well as the generative capacity to reconstruct images, not only those presented during training but also those never presented before (Fig 2C) indicate that the model had learned the underlying statistical regularities of naturalistic images and developed latent representations thereof.

### Robustness against noise

**Fig 3:**
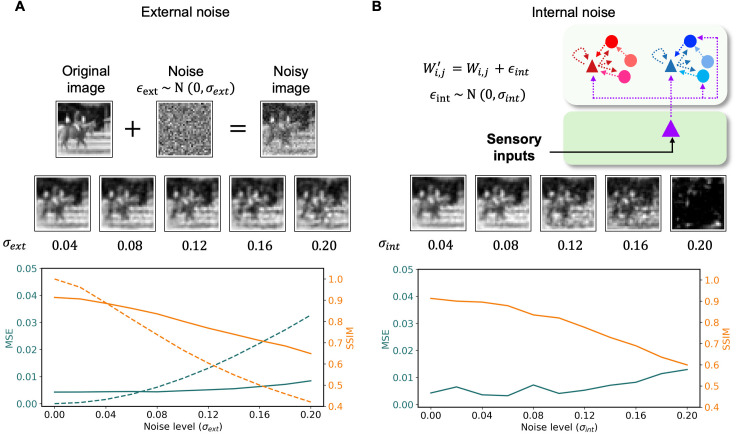
Robustness against noise. The robustness of reconstruction performance was tested against two types of noise: (A) external noise, $\in_{ext}\sim \;N\left(0,\;\sigma_{ext}\right)$, was added to the sensory input; (B) internal noise, $\in_{int}\sim \;N(0,\;\sigma_{int})$, was added to synaptic weights with Gaussian noise. Despite decreases in SSIM and increases in MSE values, the model was able to infer the causes of sensory inputs up to high levels of external and internal noise. Reconstructed images with different levels of noise are shown in the middle of each panel. Orange lines indicate the structural similarity index measure (SSIM). Dark teal lines indicate the mean squared error (MSE). Solid lines compare between original images and the images reconstructed from the noisy images, whereas dotted lines compare between the original and noisy images. Note that we did not compare between the original and noisy images when adding internal noise, as it has no effect on images themselves.

To examine whether our architecture can acquire a robust internal model that can withstand a moderate level of sensory noise or synaptic weight jitters, we introduced Gaussian noise either to input images (i.e., external noise; Fig 3A) or to synaptic weights within each microcircuit (i.e., internal noise; Fig 3B). It is important to clarify that neither of these noise types affect the learned representation itself. Given that the objective of our model, and of predictive coding in general, is to accurately infer sensory inputs by minimizing prediction errors, the low MSE (mean squared error) and high SSIM (structural similarity index measure, reflecting perceptual similarity beyond pixel-level comparisons) values demonstrate the model’s robustness, showing its ability to maintain adequate performance despite noise (Fig 3), which is important given the presence of noise in real brain networks.

When presented with images with external noise, the reconstructed images matched the original images better than the actual noisy inputs it had been provided with (solid orange line above dotted orange line, SSIM_recon_ > SSIM_input_ for noise levels > 0.05, and solid dark teal line below dotted dark teal line, MSE_recon_ < MSE_input_ for noise levels > 0.06; Fig 3A). The low MSE values and high SSIM values up to 16% of noise level to within-circuit synaptic strengths suggest that the model was also able to withstand a moderate level of synaptic jitter (Fig 3B), which is a salient feature of biological neural networks reflecting internal synaptic heterogeneity [42].

### Emergent oscillations

**Fig 4:**
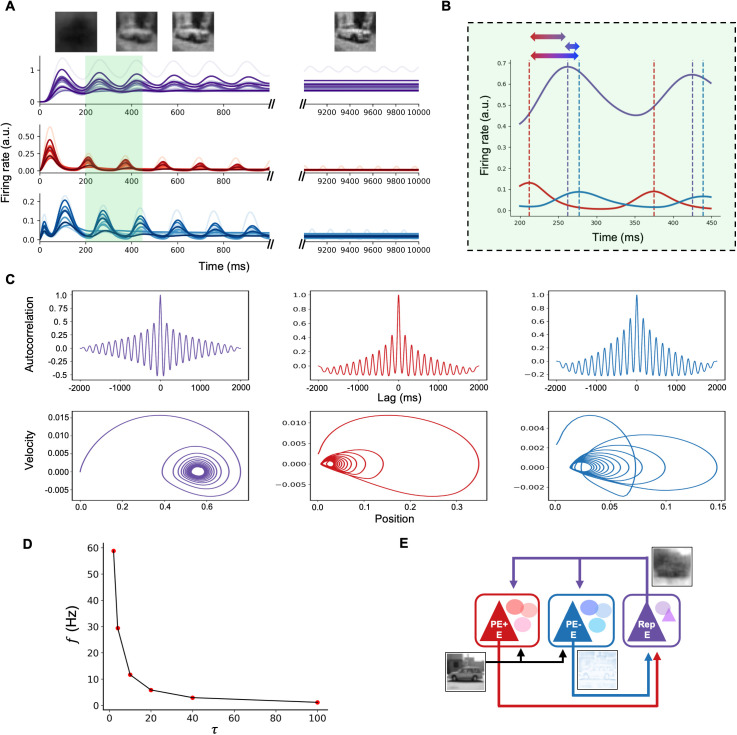
Emergent oscillations in population activity. (A) The population activity of excitatory cells in representation (purple line; top panel), positive (red line; middle panel) and negative (blue line; bottom panel) prediction error microcircuits exhibited rhythmic activities in response to naturalistic images. As prediction errors decrease, the activity of representation microcircuits converges to generate stable images, thereby yielding better reconstructions (top images) of incoming sensory inputs. (B) The highlighted area in (A) is enlarged to show that phase differences between the two prediction error microcircuits (bottom bidirectional arrow; red to blue dotted lines), negative prediction error and representation microcircuits (middle bidirectional arrow; purple to blue dotted lines), and positive prediction error and representation microcircuits (top bidirectional arrow; red to purple dotted lines) remain constant across time. (C) Autocorrelation and phase portraits of the excitatory cell population responses indicate damped oscillatory activity with stable fixed points. Colors as in (A). (D) The frequency of these oscillations is dependent on the membrane time constant (τ). (E) The oscillatory dynamics reflects the inference process of predictive coding, wherein hierarchical interaction between two prediction error (PE+ and PE-; red and blue, respectively) in area 1 and representation microcircuits in area 2 (purple) minimizes the discrepancy between actual and predicted sensory signals. The grayscale image of a car (bottom left connected to black arrows) represents sensory inputs, the blue image of the car (bottom right above the blue arrow) depicts the negative prediction error signals in Area 1 (positive counterpart not shown for brevity), and the ‘blurry‘ image of the car (top right next to purple arrows) depict the prediction of sensory inputs projecting from Area 2 to 1. Note that the reconstructed image is blurrier than the sensory input, because we want to show prediction error signals. After a full iteration of inference steps, the image will become clearer.

A close inspection of neural activity at the population level revealed that oscillatory dynamics emerge in prediction error and representation microcircuits. As prediction errors decreased (red and blue lines; Fig 4A), stable latent representations of sensory inputs were formed (purple line; Fig 4A) and coherent predictions of incoming sensory inputs were generated (see images on top; Fig 4A). The rhythmic activities between the three microcircuits were phase shifted (Fig 4B): the phase differences remained constant across time.

The oscillating autocorrelation plots and inward spiralling phase portraits provided quantitative evidence for the presence of oscillations in all three microcircuits (Fig 4C). The frequencies of rhythmic activities were approximately 6 Hz for representation and positive and negative prediction error microcircuits. The decreasing amplitude across lags in autocorrelation plots suggests that these oscillations were dampened across time towards stable focuses shown in phase portraits, indicating the system’s convergence towards an equilibrium state where prediction error is minimized and representations stabilize. While the frequency of oscillations observed from microcircuits occurred in alpha [43] and/or theta [44,45] range, this largely depended on the membrane time constant of neurons (Fig 4D). The shorter the membrane time constant was, the faster the oscillation frequency became.

The rhythmic activity observed in PC microcircuits (both during training and testing phases) and their dampening oscillatory dynamics suggest that the oscillations at the population level found in our simulations are temporal signatures of the inference process of predictive coding (cf. [31]). More precisely, the oscillations in our model are generated by cortico-cortical interactions between excitatory cells of prediction error and representation microcircuits (Fig 4E; see S1 File for example), going beyond the simplified chain-like descriptions provided in previous studies [31]. All three interneurons in both PE microcircuits also showed dampening oscillatory dynamics (see S1 Fig).

### Oddball experiment

**Fig 5:**
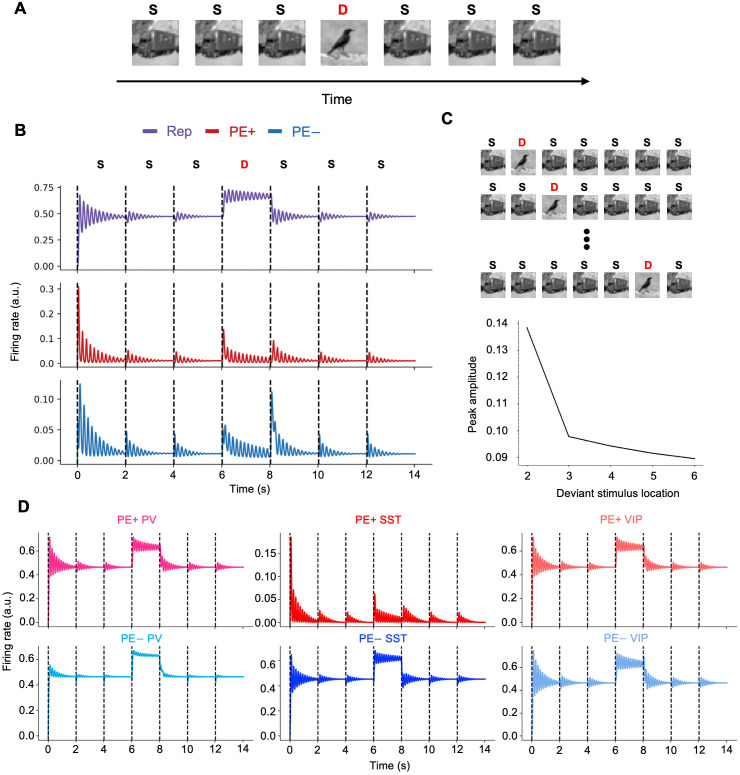
Oddball paradigm. (A) A typical oddball paradigm, in which standard stimulus, S, is repetitively presented, interrupted by a deviant stimulus, D. (B) Population activities of L5 excitatory neurons in representation (Rep; purple line) and L2/3 excitatory neurons in prediction error microcircuits (PE+ and PE-; red and blue lines) in response to the oddball sequence in (A). Prediction errors decreased over time as a consequence of the inference process. Repetitive presentation of the standard stimulus (e.g., a truck image; S) resulted in lower initial prediction errors and faster error minimization compared to the initial encounter with the stimulus (0-2 s; red and blue lines). Similarly, steady-state latent representations were reached more rapidly with repetitive presentation of a stimulus (Rep; purple line). Meanwhile, a deviant stimulus (e.g., a bird; D) elicited increased neural activity in prediction error microcircuits and led to a different steady-state representation. (C) Varying the location of deviant stimulus in the oddball sequence led to differences in initial peak amplitudes of responses in prediction error microcircuits. (D) Responses of interneurons to the oddball sequence used in (A).

When presented with a sequence of repetitive stimuli, interrupted by a deviant stimulus (oddball paradigm; Fig 5A), population activities of excitatory neurons in representation microcircuit and both positive and negative error microcircuits showed an oddball effect (Fig 5B), viz. an enhanced firing rate (on top of the dampened oscillation).

The initial standard stimulus evoked a large transient response in L5 representation and L2/3 prediction error neurons (Fig 5B), as the network settles from zero activity. Subsequent standard stimuli elicited smaller responses from the resulting non-zero network state. Reflecting predictive coding principles, these smaller responses correspond to reduced initial prediction errors that are minimized more rapidly (red/blue lines); concurrently, latent representations also stabilized more quickly (purple line) after the initial inference. These dynamics, including network oscillations, stem from the iterative interaction between L5 representation and L2/3 prediction error circuits. As the network converged, improving predictions reduced prediction error signals (L2/3 activity), which in turn dampened network oscillation amplitude. Oscillatory power is therefore linked to this interactive error minimization process. Finally, the deviant stimulus disrupted this state, increasing prediction error activity (red/blue lines) and driving renewed representation activity (purple line), consistent with a mismatch response.

The ratio between inter-stimulus interval (ISI) and neuronal time constant ($\tau_{\text{exc}}$) had a large impact on the oddball effect: when this ratio was less than 1, the effect was more prominent than when greater than 1. The peak amplitude of prediction error activity was highest when the deviant stimulus was presented earlier in the sequence than later (Fig 5C).

### The role of interneurons

To systematically investigate the role of interneurons in PC of sensory inputs, we set the neural activity of each of the three interneurons to zero (i.e., ${\text{r}}_{\text{i}}=0$ where r represents firing rate of a neuron and i∈{PV, SST, VIP}), simulating an optogenetic silencing experiment. We analyzed population activities of the positive and negative prediction error microcircuits and the network’s performance on reconstruction of input images.

**Fig 6:**
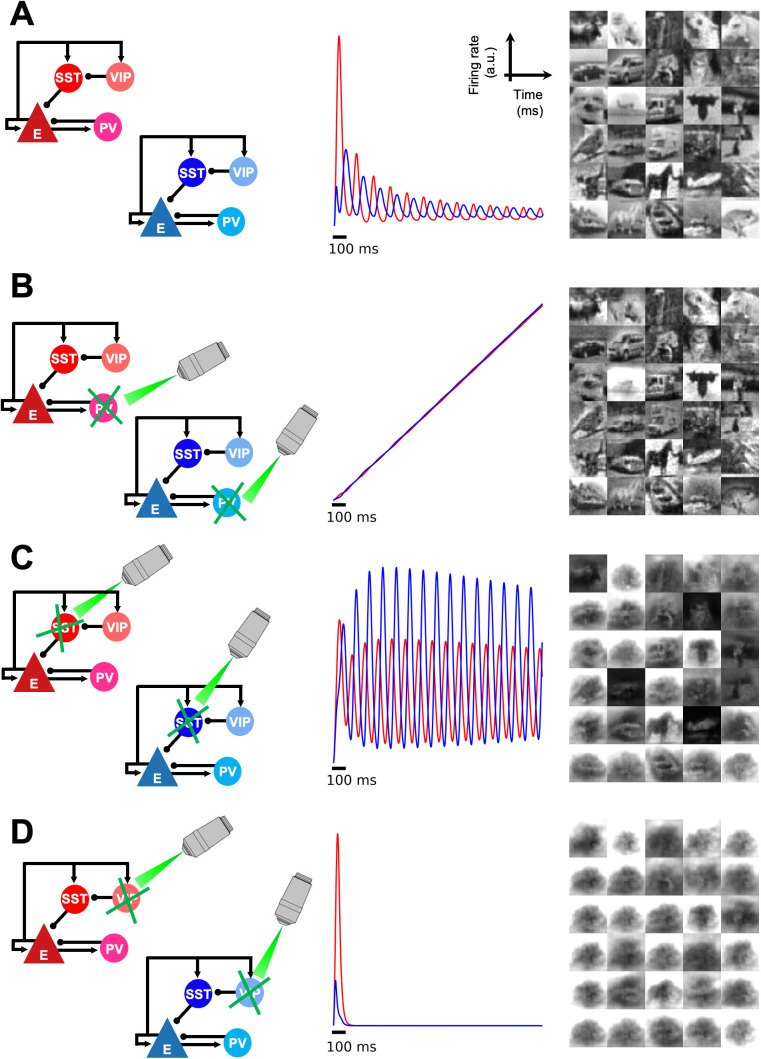
Optogenetic simulation to silence interneurons. Each of the three types of interneurons (B: PV, C: SST, and D: VIP) was silenced via optogenetic simulation to investigate their roles in predictive coding of naturalistic images. For each subpanel, the corresponding optogenetic simulation is depicted on the left, mean firing rates of excitatory cells in positive and negative prediction error (PE) microcircuits (red and blue, respectively; in arbitrary units) is shown in the middle, and reconstructed images (i.e., predictions of sensory inputs) are shown on the right. (A) A canonical microcircuit without deactivating any interneuron is presented as a reference for comparison. Positive and negative PE responses decrease across time as a result of perceptual inference. The rhythmic activities of the two PE microcircuits were phase-shifted (see Fig 4B). (B) When PV cells were silenced, both positive and negative PE microcircuits lacked inhibitory inputs, leading to a constant increase in prediction errors. However, representation microcircuits were still able to make predictions about incoming sensory inputs, thanks to the inadvertent substitute loop that still retains the capacity to predict sensory inputs, in which the positive PE microcircuit relays bottom-up sensory inputs and the negative PE microcircuit transmits top-down predictions. The representation microcircuit integrates these two signals to update internal representations of sensory inputs. (C) Without SST cells, both positive and negative prediction error microcircuits showed persistent rhythmic activities throughout the duration of stimulus presentation, resulting in a failure to minimize prediction errors and predict incoming sensory inputs. (D) Knocking out VIP cells silenced both types of prediction error microcircuits and led to a failure to reconstruct images. For the image reconstructions in the rightmost column, the final Mean Squared Error (MSE) values were: 0.00538 (control), 0.0132 (PV-silenced), 0.0711 (SST-silenced), and 0.223 (VIP-silenced). The corresponding Structural Similarity Index Measure (SSIM) values were 0.923, 0.847, 0.521, and 0.311.

Silencing PV cells (left; Fig 6B) resulted in a continuous increase of excitatory firing rates in both the positive and negative prediction error circuits, thereby shutting down the ~ 6 Hz rhythm (middle; Fig 6B). Note that, in a more biologically realistic scenario, saturation mechanisms would place an upper bound on this firing rate increase. While inhibiting PV activities within the two PE circuits disrupted the prediction-error feedback loop between L2/3 PE and L4/5 Rep circuits, it inadvertently created a substitute loop that let the network retain the ability to predict sensory inputs. Instead of propagating positive and negative PEs, the two PE circuits simply ended up relaying bottom-up sensory inputs and top-down predictions to L4 excitatory and PV cells of Rep circuit (black triangle and blue circle in Area 1; Fig 1C). Integrating these activities led to a proper update of representations.

While the impact of PV silencing on final reconstruction accuracy appears minor (Fig 6B, right panel), the significant reduction in oscillatory power (Fig 6B, center panel) warrants careful interpretation, particularly concerning the role of oscillations in the circuit. These oscillations are not merely epiphenomenal; as established earlier, the causal interactions between representation and prediction error neurons mediating the inference process inherently manifest as oscillatory dynamics in our model. Any alteration, such as reduced PV activity, thus has causal effects on how these interactions unfold over time.

The specific reason for the relatively small impact on final prediction accuracy under PV silencing stems from targeting PV cells in both positive and negative prediction error microcircuits simultaneously. This leads to reduced inhibition and consequently higher firing rates in both sets of excitatory error neurons, whose influences on the overall prediction tend to cancel each other out regarding the final static outcome. However, this manipulation fundamentally disrupts the balanced E-I dynamics crucial for the oscillatory interactions themselves, leading to the observed substantial reduction in oscillatory power (Fig 6B, center panel). This finding is consistent with observations in schizophrenia research, where reduced PV cell function or expression [46,47] is linked to decreased gamma-band oscillations [48]. This means that inactivating a given cell type could not hurt the performance of the inference process but still alter the underlying dynamics.

Despite silencing SST cells (left; Fig 6C), oscillatory activities were still observed in both the positive and negative prediction error microcircuits (middle; Fig 6C). However, these oscillations were not dampened to a fixed point. This indicates a failure to correctly infer the causes of sensory inputs, as evidenced by the model’s inability to reconstruct input images (right; Fig 6C). In the absence of VIP cell contributions to prediction error computation (left; Fig 6D), excitatory cell activity in both the positive and negative prediction error microcircuits quickly shuts down (middle; Fig 6D). Removing the VIP-SST disinhibitory circuit shifted the excitatory-inhibitory balance in synaptic inputs towards inhibition of excitatory neurons, resulting in a cessation of firing activity. Without the feedforward prediction errors that guide the proper inference of incoming sensory inputs, the network was unable to reconstruct input images (right; Fig 6D).

## Discussion

In this work, we have demonstrated that PC may be explained in the context of a biologically plausible set of principles of cortical architectures. We have attempted to minimize the number of functional assumptions and instead root our model on neurobiologically-compatible principles (Fig 1): hierarchically-organized relationships between pairs of cortical areas, in terms of laminar projection patterns [29], laminar organization of microcircuits [6,16,28], Dale’s principle for excitatory and inhibitory cells [49], diversity of cell types [25,27,50] and Hebbian learning rules [51].

Functionally, the model implements predictive coding computations inspired by the canonical computational model, i.e., considering prediction errors as the Gaussian likelihood whose log-likelihood is the squared-error criterion [2]. Top-down predictions (L5 excitatory activity) are compared against bottom-up inputs (via L4), with L2/3 excitatory firing rates representing the resulting prediction errors. The iterative, error-driven refinement of the internal model aligns conceptually with theoretical works emphasizing such mechanisms [52,53]. However, as opposed to abstract neural populations often used in PC models, our model makes a distinction in that it explicitly incorporates distinct cortical cell types (E, PV, SST, VIP) and their interactions within a simplified layered cortical microcircuit. This allows us to explore how specific interneuron dynamics contribute to prediction error processing, providing a more biologically detailed account.

Likewise, our model is able to perform PC efficiently (Fig 2), but the results go beyond simply inferring the sensory input towards more neurobiologically relevant directions: good performance in the presence of external and internal noise (Fig 3), division between positive and negative prediction error circuits [12,14,26,27], emergence of neural oscillations during visual processing (Fig 4) [31,54–57], and enhanced neural responses to deviant stimuli in mismatch negativity and oddball paradigms (Fig 5) [32–40] (see [58] and [11] for computational model and theory). Importantly, this biological plausibility is further supported by our model’s microcircuit, which incorporates several key connectivity patterns observed in studies such as [59].

Our model constitutes a gross simplification of the real complex circuitry in real cortical networks, as compared for example with recent neuroanatomical studies highlighting a rich structure between E, PV, SST and VIP cells [59]. Still, our computational description incorporates several key connectivity patterns consistent with these biological observations. For example, our model’s strong excitatory connections between L2/3 excitatory and PV cells and the disinhibitory circuit from VIP to SST to excitatory cells in prediction error microcircuits align with the high connection probabilities and strengths reported in [59]. Similarly, the high connection probability from i2/3Htr (VIP) to i2/3Sst (SST) (0.625) supports our disinhibitory circuit. Although [59] report relatively weak projections from layer 4 to neurons in layer 2/3 (possibly due to their focus on connectivity patterns within a 75 μm radius), our model also considers these projections, which constitute the basis for columnar and canonical microcircuits [6,60,61]. Likewise, projections from excitatory to VIP cells were included since VIP cells are often the target of glutamatergic input which triggers disinhibitory pathways also included in our circuits [41], and this allows us to consider lateral inputs beyond more limited neuroanatomical estimations. Finally, we have considered that all existing connections have a similar strength (equal to one, in the model). This is a necessary limitation for a first step in the study of biologically plausible implementations of PC, but it should be extended in future studies by including synaptic heterogeneity (and potentially also plasticity) in those connections.

Consistent with the biologically realistic microcircuit architecture described above, the consideration of multiple types of inhibitory neurons constitutes a fundamental ingredient in our study, and it follows previous work on the role of SST and VIP cells in generating prediction errors [23,25]. A large number of studies in recent years have highlighted SST and VIP cells as key players in integration of bottom-up and top-down input [30,60,62–65], thus their involvement in PC should be seriously considered. Connections between E, PV, SST and VIP cells were chosen following canonical circuit configurations common in the literature [62–64], which include important connectivity patterns to reflect the role of inhibitory neurons in providing blanked inhibition and also disinhibitory pathways. Patterns of external projections to those neurons were, on the other hand, determined via a combinatorial search. In such a search, we indeed found different connectivity patterns which would give rise to the same behavior, which suggests some level of degeneracy –allowing for multiple connectivity combinations to yield the same performance when computing prediction errors. This level of degeneracy could be actually much higher if we relax our constraints on the microcircuit connectivity, allowing for example weaker connections to excitatory cells which could be compensated with stronger external projections. This suggests that cortical circuits with the same functionality could indeed be formed via different mechanisms and display a diversity in circuit motifs [23].

Furthermore, our results align with experimental findings on the role of inhibitory interneurons. The hierarchical framework described in predictive routing models [66], where top-down signals modulate VIP cells influencing SST and E activity, is reflected in our model where such modulation impacts local activity and oscillatory power via VIP-SST interactions. A recent finding on VIP cells exerting local control on gamma gain and coherence through disinhibition [67] is also consistent with our model; optogenetic silencing of VIP neurons diminished oscillatory patterns, reflecting disruption of their disinhibitory effect on SST cells and supressing local oscillations. This disruption of local VIP-mediated control may impact the coordination between prediction error and representation circuits across the hierarchy. Meanwhile, our results of increased local oscillatory power upon SST suppression suggest a complementary role for SST cells in local power regulation within prediction error processing, distinct from their role in global synchronization [68]. In our model, oscillations directly represent prediction error minimization, and SST cells dampen these oscillations as prediction errors are resolved. Suppressing SST cells removes this dampening effect, causing oscillations to persist and amplify, which impairs the model’s predictive accuracy.

Contrary to the diversity of cell types, neural oscillations were not specifically ingrained in our architecture, but rather constitute an emergent phenomenon resulting from neural dynamics due to excitatory-inhibitory interactions within microcircuits. The nature of these oscillations, of the stable-focus type or ‘damped oscillator’ type, does not constitute a problem by itself. While these oscillations will decay and disappear in an idealized system, the presence of external or internal noise is enough to maintain such oscillations in a noise-driven state and propagate them to other areas within the cortical hierarchy, as shown elsewhere [69–71]. Indeed, the performance of our model is well preserved under low or moderate levels of external or internal noise (Fig 3), so noise-driven oscillations are likely to survive in real conditions. Furthermore, fast oscillations such as gamma, beta, and perhaps even high alpha might be better described by noise-driven oscillations (i.e., stable focus under noise) rather than by a sinusoidal-like limit cycle, given the low amplitude and weak coherence observed in LFP recordings for these ranges [55,72,73]. As reflected by our simulations, different interneuron types will likely play complementary roles in these oscillatory dynamics, which makes the question experimentally accessible via optogenetic inactivation protocols. While the precise frequency of the oscillations in our model depends on parameters such as population time constants, whose values are not directly accessible from recordings, the potential range includes frequencies from gamma to alpha. However, the current model was not explicitly designed to replicate the distinct frequency-specific signaling roles (e.g., gamma vs. alpha/beta) proposed in some frameworks, such as predictive routing models [66]. Our focus was on establishing the core PC mechanisms within a plausible circuit, where oscillations emerged naturally. The latter case would be in line with previous computational work which has linked the presence of top-down signals with the appearance of alpha oscillations [71] and particularly in the context of predictive coding [31]. What the exact role of these oscillations is within PC is a question that needs further study, although our results already suggest that they might be reflecting, or mediating, the communication between representation and error neurons during the inference process. This hypothesis is at least consistent with experimental observations of alpha and theta waves mediating inter-regional communication [74,75]. Alpha waves, for example, are known to propagate in the feedback direction in the absence of stimuli [72,75–78] which could very well involve the interaction between error and representation units in our model. Theta oscillations might play a similar role, although this is less clear given that their propagation has often been linked to feedforward traveling waves in the brain [75,78]. Meanwhile, [79] limits the role of oscillatory dynamics in predictive processing to stabilizing neural representations and facilitating plasticity whilst arguing that aperiodic transients are responsible for sensory inference.

Further clarifying the link between specific mechanisms and observed phenomena like MMN is also important. While aspects of MMN can potentially be explained by mechanisms such as stimulus-specific adaptation [80], our model demonstrates that MMN-like responses naturally emerge within a predictive coding framework involving interactions between representation and error circuits (Fig 5). Although our aim was not to definitively arbitrate between mechanisms, a predictive coding framework may provide a more comprehensive explanation for key MMN features, particularly for the case of high cortical areas like prefrontal cortex. For example, [81] demonstrate that MMN reflects prediction error signals generated within cortical microcircuits, showing that its subcomponents are better explained by prediction error than adaptation. Recent work [32] further support this by showing that neuronal mismatch responses in the medial prefrontal cortex are better explained by prediction error signaling. Finally, [82] dissect MMN into early and late subcomponents in frontal and centro-frontal cortex, showing that both detect deviants in local and global sequence regularities, a feature that is difficult to explain through adaptation alone. This posits PC as perhaps a plausible mechanisms at least for higher cortical areas like prefrontal cortex.

To further probe the role of predictive mechanisms in MMN generation within our framework, we conducted an additional simulation reducing top-down inputs, guided by experimental work on propofol-induced loss of consciousness [83]. Simulating this reduced top-down influence (by decreasing the effective weights of L5 projections to L2/3 prediction error circuits, see S4 Fig), we observed diminished MMN-related oscillations, consistent with the observation of propofol-induced alpha/beta reduction in the sensory cortex, suggesting a disruption of normal top-down inhibitory control and hierarchical predictions. Crucially, when stimuli were omitted under these simulated conditions, mismatch activity was primarily generated by the negative prediction error circuit (S4 Fig). This selective response pattern—specifically, the generation of a negative prediction error signal indicating an unfulfilled prediction even without bottom-up input—provides strong support for interpreting MMN within our model as a signature of predictive coding, rather than solely stimulus-specific adaptation.

Our model can, and should, be extended in different directions in the future. First, while we have committed to keep our working assumptions close to neurobiology, there are several aspects that could still use refinement. For example, neural dynamics has been described in terms of mass models of population activity, but spiking network models for PC can be used to further enhance realism [14]. In addition, cortical microcircuits with E, PV, SST and VIP cells are idealizations, and deep layers have been modelled with less detail than superficial ones. For instance, layer 5 pyramidal cells in Area 2 simply relay top-down predictions in our model. Although this is an important role within the proposed mechanism (and provides an interesting prediction on the effect of optogenetically silencing layer 5 neurons), it could be argued that deep layers might have a more complex participation in PC. The structural mimicry of anatomical projection patterns in our model therefore foreshadows future development of the model to incorporate more biologically grounded elements of deep layer functionality as and when they are determined by further research. These modelling limitations could be solved or alleviated by heading towards data-constrained models of cortical columns, for which several models with multiple interneurons are now available [59,60,65,84]. Likewise, the laminar-specific connectivity patterns should be improved once more detailed information becomes available. This is particularly important as it is known that the laminar specificity of inter-areal connections varies across species –for example, top-down projections tend to stem from infragranular layers in primates [29], but the pattern is less clear in rodents [85]. It is possible that these deviations could be compensated by connectivity properties of local circuits, but this issue deserves nonetheless more attention.

Another simplification in our model lies in its image processing. While our model simplifies image processing by employing a pixel-by-pixel representation, and does not explicitly implement lateral effects such as surround suppression, a function where SST neurons are known to play a critical role, it allows us to focus on the core dynamics of predictive coding and learning, investigating how a hierarchical predictive coding model can learn to accurately predict incoming sensory inputs, represented as individual pixel values, even with simplified connectivity. To elaborate: area 1 encoded inputs at the pixel level (resulting in single-pixel receptive fields) and projected with full convergence to area 2 neurons (yielding full-field receptive fields; see S2 Fig for details). Despite contradicting biological retinotopy and complex receptive fields, this non-topographic structure was chosen specifically to enable a focused study of core predictive coding mechanisms by simplifying the complex spatial processing components. Furthermore, regarding the use of image reconstruction, it is important to acknowledge that successful pixel-by-pixel reconstruction does not equate to the full richness of biological perception, which involves higher-level feature extraction and interpretation. In the context of our model, reconstruction served primarily as a quantitative proxy to evaluate whether the latent representations learned in Area 2 captured sufficient statistical information from the input to allow for its regeneration. The model’s ability to perform this task suggests that these learned representations, while developed within a simplified setting, are indeed relevant for perceptual inference and form a foundation upon which more complex perceptual functions could potentially be built. Importantly, even in this simplified scenario, SST cells contribute to the modulation of prediction errors. Specifically, our model demonstrates that SST neurons carry top-down predictions, allowing for the adjustment of prediction error signals based on pixel-level discrepancies. This mechanism can be viewed as a form of gain modulation, where prediction error signals are weighted and adjusted based on pixel-level discrepancies.

Further extensions could involve considering other learning rules on top of these circuits, such as inhibitory plasticity rules, which could contribute to balance excitation and inhibition and better refine prediction error circuits [23]. Our model also assumes that the learned feedback weights (the purple arrows from L5 excitatory cells in Area 2 to L2/3 PE circuits in Area 1; Fig 1C) targeting positive and negative prediction error circuits are approximately the same, even though they target different neurons. While this condition may be in practice relaxed in the model and good results are obtained when these weights are moderately different, it is necessary to explore which concrete biophysical mechanism might be able to provide this soft homeostatic effect. As positive and negative prediction error circuits might coexist within the same space [27], local neuromodulation stands as a plausible element here. Finally, beyond adding more neurobiological features to the model such as the aforementioned topographic connectivity and more varied receptive field structures, another open avenue is to expand the functionality of the model to implement more sophisticated types of task, including object invariance properties to take into account visual rotations or transformation common in real-world applications [7].

Overall, the present work illustrates that PC does not require a hypothesis-driven implementation (such as a free energy minimization principle), but rather that it can be obtained by considering realistic (or at least neurobiologically-informed) cortical architecture, cell variability and Hebbian learning rules. In this context, while some conceptualizations of PC involve top-down predictions informed by high-level priors derived from accumulated prior experience (akin to memory stores; [86]), our model implements priors implicitly through synaptic weights adapted via Hebbian learning (Eq. 2). These priors, representing the statistical regularities of the sensory environment, become encoded within the synaptic weights connecting representation and prediction error circuits. Therefore, the backward projections embody the network’s current hypothesis or ‘best guess’ about the input, based on its learned internal model encoded in these weights. This implementation aligns with the core PC principle of generating predictions based on learned regularities to minimize prediction errors. According to our results, PC can emerge naturally in realistic cortical networks without additional assumptions. Our finding that oscillations emerge spontaneously in our model demonstrates a link between PC and brain oscillations tied to cortical bottom-up and top-down processing, underscoring its neurobiological plausibility.

## Methods

### Network model

Neurons in our network follows a linear firing rate model [87]:


$$\tau_{\text{k}}\;\frac{\partial {\text{r}}_{\text{i}}}{\partial \text{t}}\;=\;-{\text{r}}_{\text{i}}+f\left(\sum {\mathrm{\backslash nolimits}}_{\text{j}}{\text{W}}_{\text{ij}}\;{\text{r}}_{\text{j}}\;\right)$$ (1)

The firing rate of a neuron *i* ($r_{i}$, where i ∈{E, PV, SST, VIP}) changes according to the membrane time constant ($\tau_{\text{k}}$, where k∈{exc, inh}), the leak term ($-{\text{r}}_{\text{i}}$), and the integration of incoming synaptic inputs ($\text{f}(\sum_{\text{j}}{\text{W}}_{\text{ij}}\;{\text{r}}_{\text{j}})$) (Eq. 1). The membrane time constants for excitatory and inhibitory neurons were kept the same (20 ms) for simplicity. The activation function (f) was rectified linear unit (ReLU).

Note that while neuron types are labelled based on their circuit roles and key connectivity patterns (E, PV, SST, VIP), the underlying model neurons are simplified representations without detailed biophysical considerations such as specific morphology or spiking dynamics, as we choose to focus on the circuit-level mechanisms and emergent network dynamics within the predictive coding architecture.

Our network is constituted by two cortical areas (labelled as Area 1 and Area 2). Area 1 contains two components, one for layer 2/3, which is in charge of the prediction error computation, and another for layer 4, which is receiving bottom-up sensory input and projecting it to layer 2/3. Layer 4 contains 1024 excitatory neurons, corresponding to each of the pixels in the input, while layer 2/3 contains two blocks of 1024 microcircuits, one for the positive prediction errors and the other for the negative prediction errors. These microcircuits, which contain four different neural mass units corresponding to E, PV, SST and VIP cells, is detailed in the subsection below. The connectivity from layer 4 neurons to layer 2/3 microcircuits is one-to-one. Area 1 does not explicitly include layer 4 PV interneurons (for simplicity of the sensory pathway) or layer 5 excitatory neurons, because we do not include feedback projections from the primary visual cortex to the thalamus in our model.

Area 2 contains one component, which involves E and PV interneurons in layer 4 and also E neurons in layer 5, with one-to-one connectivity between them. These circuits encode the representations that project top-down predictions to Area 1 in our network. Layer 2/3 microcircuits are not included in Area 2 since our network does not project further away in the cortical hierarchy, but they could be easily considered as illustrated in Fig 1C. We have 784 neurons for each of the three types in Area 2. Projections between Areas 1 and 2 are densely connected (i.e., displaying all-to-all connectivity), although they are regulated by synaptic plasticity rules (see Fig 2 and S1 Table).

In such a hierarchy, residual prediction errors ascending the cortical hierarchy signal discrepancies between top-down predictions and lower-level representations. Functionally, these errors convey abstract mismatches, driving the higher-level areas to form compressed representations that capture higher-order relationships rather than raw sensory details. This mechanism embodies the cortical compression gradient [88], which is explicitly implemented in our model through dimensionality reduction (e.g., projection from 1024 Area 1 prediction error units to 784 Area 2 representation units, which corresponds to the feedforward projection from layer 2/3 excitatory neurons in Area 1 to agranular higher order cortex, specifically to layer 4 excitatory neurons in Area 2).

### Microcircuit configurations

The connectivity pattern among the four cortical cell types in positive and negative prediction error microcircuits and the synaptic input patterns to those neurons was determined via an exhaustive combinatorial search. In particular, we aimed to identify a canonical microcircuit consisting of four cortical neuron types (E, PV, SST, and VIP) and capable of generating both positive and negative prediction errors. The direction of the error (positive or negative) is determined by the relative strength of bottom-up (L4 E) and top-down (L5 E) inputs. To identify some of the possible circuits fulfilling these conditions, we conducted an exhaustive combinatorial search across n = 32,768 potential combinations. This search explored all possible within-circuit connectivity and synaptic input patterns, aiming to identify configurations compatible with established neurobiological principles.

**Fig 7:**
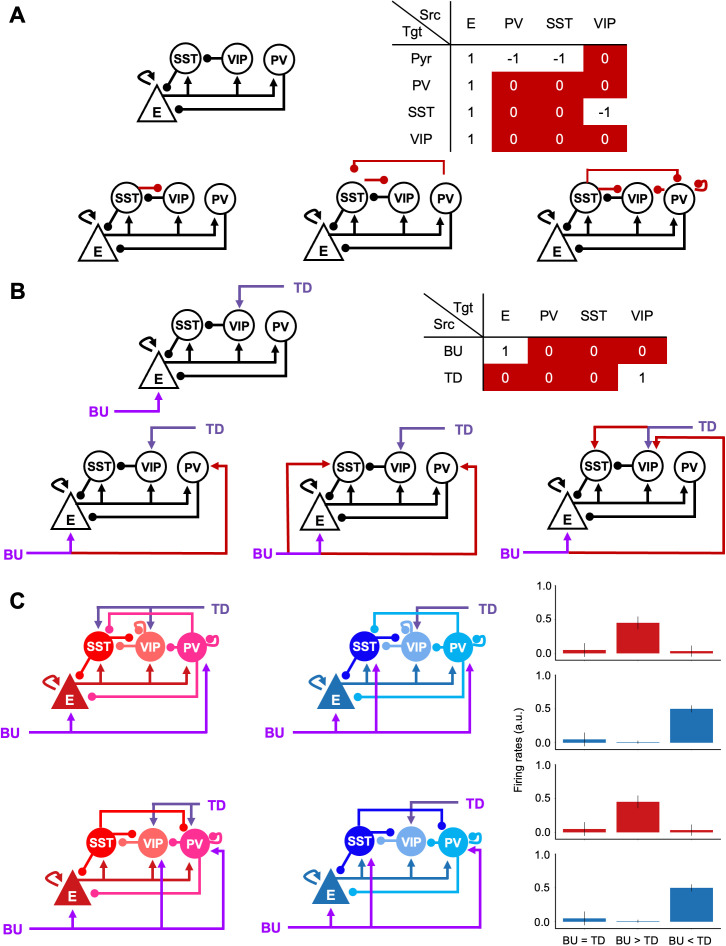
Combinatorial search for canonical prediction error microcircuits. An exhaustive combinatorial search (n = 32768) explored all possible within-circuit connectivity and synaptic input patterns. (A, B) Tables illustrate the variable connections (red) and fixed, biologically grounded constraints (non-highlighted) used in the search, along with three example circuit configurations. Each combination was evaluated across three scenarios (BU = TD, BU > TD, BU < TD), as depicted in Fig 1A. (C) Two example microcircuit configurations from the 173 combinations that successfully generated both positive and negative prediction errors.

To constrain the search space, we imposed the following biologically grounded constraints, based on empirical findings [6,16,41]: 1) excitatory neurons receive bottom-up sensory inputs; 2) VIP cells receive top-down inputs; 3) SST and PV cells provide inhibitory input to excitatory cells; 4) VIP cells disinhibit excitatory cells by inhibiting SST cells; and 5) excitatory cells project to all cell types within the microcircuit. These constraints defined a template within-circuit connectivity pattern (see upper panels in Fig 7A and 7B). We then varied the remaining connections (red-highlighted cells in tables of Fig 7A and 7B) and synaptic input patterns, resulting in 512 connectivity and 64 input patterns.

Each combination was evaluated across three scenarios (Fig 1A): 1) equal bottom-up and top-down inputs; 2) stronger bottom-up inputs (negative prediction error); and 3) stronger top-down inputs (positive prediction error). We selected combinations that exhibited no prediction error in scenario 1, but generated opposite prediction errors (positive/negative) in scenarios 2 and 3, depending on the input pattern. This search yielded 173 successful combinations, each consisting of a within-circuit connectivity pattern and two input patterns for positive and negative prediction errors (Fig 7C).

For our study, we randomly selected one of these 173 combinations (Fig 1A) to illustrate the general principles of prediction error computation within our model. This selection was made without loss of generality, as any of the 173 combinations would have produced qualitatively similar results (Fig 7C).

### Learning naturalistic images

Only a small subset of the CIFAR-10 dataset (nimage = 2560, nclass = 10, and nsample = 256; 5.1% of the total images in the dataset) was used for the training. Without loss of generality, with respect to predictive coding of sensory signals, the colored images of the CIFAR-10 dataset was converted to grayscaled images to reduce the computational costs.

Each neuron in the input area (L4 E cells in Area 1) received constant input (${\text{X}}_{\text{i}}$), the magnitude of which matches a corresponding pixel (${\text{X}}_{\text{i}}$ ∝ px) in an image in a one-to-one fashion (a single pixel value to each L4 E cell; i.e., $\tau_{\text{k}}\frac{\delta {\text{r}}_{\text{i}}}{\delta \text{t}}=-{\text{r}}_{\text{i}}+\text{f}({\text{X}}_{\text{i}})$). For instance, a darker pixel has a value close to 0.0 and a lighter pixel has a value close to 1.0 (for more details, see S2 Fig).

To reconstruct an image, L5 E cells in Area 2 neurons make projections (i.e., top-down predictions) to prediction error microcircuits in area 1 ($\text{W }{\text{r}}_{\text{r}}^{\text{Rep}}$). Note that the reconstruction of an image is only for intuitive (or semantic) purpose, and real brain circuits would likely follow this principle to reconstruct more abstract neural activity patterns. We opted for dense connections to better facilitate the implementation of biologically plausible Hebbian learning mechanisms and, critically, to allow for the implementation of predictive coding in a cortical column by incorporating interneurons in cortical circuits, a key focus of our study. Alternative connectivity patterns, including sparse architectures and/or convolutional neural networks, could be explored in future work to further investigate biological plausibility.

To improve computational efficiency, we employed a standard batch processing method (visually illustrated in S3 Fig). This method, which is a computational expediency and not a claim of biological realism, involved presenting subsets of the input stimuli in parallel to multiple identical copies of the network. Once the stimulus presentation and inference had been complete within each network copy, the mean of the resulting neuronal activities were calculated across the copies. This averaged activity is then used to update the synaptic weights according to the Hebbian learning rule. With a batch size of 64 images, each image was presented for 2 seconds, during which the model formed latent representations of incoming sensory inputs. Before presenting the next batch of stimuli, the network was shown an empty screen for 0.2 seconds. During this inter-stimulus interval, neural activities across the cortical hierarchy quickly reset. The model was shown each image 100 times.

The updating of synaptic weights between prediction error microcircuits and representation microcircuit occurred immediately after the stimulus presentation had ended and followed a Hebbian learning rule as in [14]:


$$\Delta \text{W}=\;-\gamma_{\text{w}}\;\left[{\text{r}}_{\text{E}}^{\text{PE}+}\;{{(\text{r}}_{\text{r}}^{\text{Rep}})}^{\text{T}}-\;{\text{r}}_{\text{E}}^{\text{PE}-}\;{{(\text{r}}_{\text{r}}^{\text{Rep}})}^{\text{T}}\right]$$ (2)

The first term within the squared bracket represents the contribution of positive errors (PE+), whereas the second term corresponds to the contribution of negative errors (PE-; Eq. 2). Note that $\gamma_{\text{w}}$ represents a learning rate and ${\text{r}}_{\text{r}}^{\text{Rep}}$ the firing rate of L5 E cells in representation circuit.

The extrinsic weights between PE and representation microcircuits (W; dotted lines in Fig 2A) were symmetric and shared between the positive and negative PE microcircuits. The synapses within all microcircuits had a fixed weight of one and were not subject to synaptic plasticity. S1 Table shows all synaptic weights present in our model and indicates which ones were subject to plasticity.

### Performance metrics

To quantify the accuracy of image reconstruction at the end of the sensory inference process, we employed two standard metrics: MSE and SSIM.

The MSE is defined as the mean of squared pixel-by-pixel differences between the predicted image ‘P’ (generated by the model’s top-down pathway) and the actual input image ‘A’ presented to the network (indices run over the number of pixels):


$$\text{MSE}=\frac{\text{1}}{\text{n}}\;\sum {\mathrm{\backslash nolimits}}_{\text{i}=\text{1}}^{\text{n}}{\left({\text{P}}_{\text{i}}-{\text{A}}_{\text{i}}\right)}^{\text{2}}$$ (3)

Importantly, due to the design of our microcircuit, this pixel-wise prediction error is directly encoded by the activity of prediction error neurons; consequently, the overall MSE is inherently represented by the mean firing rates of the L2/3 excitatory neurons within the prediction error microcircuits during the inference process.

The SSIM assesses the perceptual similarity between two images (in this case, the reconstructed prediction ‘R’ and the original input ‘O’):


$$\text{SSIM}=\frac{\left(\text{2 }\mu_{\text{R}}\mu_{\text{O}}+{\text{c}}_{\text{1}})(\text{2 }\sigma_{\text{RO}}+{\text{c}}_{\text{2}}\right)}{\left(\mu_{\text{R}}^{\text{2}}+\mu_{\text{O}}^{\text{2}}+{\text{c}}_{\text{1}})(\sigma_{\text{R}}^{\text{2}}+\sigma_{\text{O}}^{\text{2}}+{\text{c}}_{\text{2}}\right)}$$ (4)

Where μ_x_ denotes the pixel-averaged mean of image x, σ_x_ is its standard deviation, σ_xy_ is the covariance between both images, and c_1_ =(k_1_ L)^2^ and c_2_=(k_2_ L)^2^ are two stabilization factors, with L being the dynamical range of the image and k_1_ = 0.01, k_2_ = 0.03 two parameters. SSIM is a standard measurement which combines comparisons of mean intensity, standard deviation (contrast), and covariance (structure) to provide a score closer to human perception of image quality than simple pixel differences. SSIM serves here as a complementary measure to MSE, specifically evaluating the perceived quality and structural fidelity of the final reconstructed static image after inference has converged.

We report MSE and SSIM primarily to quantify final, static image reconstruction quality (e.g., in robustness analyses, Fig 3). For analyses focused on emergent temporal dynamics, such as oddball experiment (Fig 5) or simulated optogenetic silencing (Fig 6), SSIM is unsuitable as it compares static images (i.e., predictions made after the inference had converged). Given that the dynamic prediction error (MSE) is directly represented by the L2/3 excitatory firing rates as noted above, we present these firing rates as the primary and most direct measure for these dynamic analyses, avoiding redundant MSE plots.

## Conclusion

We propose a novel cortical network model with laminar and cell-type specific architecture that can perform perceptual inference learning of sensory input. We observed rhythmic activity spontaneously emerging during propagation of predictions and errors and dissected the role of different cortical neuron types in generating prediction errors.

## Supporting information

S1 Table List of synaptic weights in the cortical column model of predictive coding. (DOCX)

S1 Fig Oscillatory behavior of interneurons in response to naturalistic images. Similar to the excitatory cells (see Fig 4), each of the three interneurons (PV, SST, and VIP) in positive and negative PE microcircuits (color as in Fig 1C) also show rhythmic activities that dampen across time to reach stable focuses.
(TIFF)

S2 FigFeedforward sensory inputs and feedback predictions in a cortical column model of predictive coding.Each L4 excitatory cell in Area 1 (light purple triangles to the left) received a constant pixel-level input (${\text{X}}_{\text{i}}$), representing a receptive field of one pixel. L5 excitatory cells (dark purple triangles to the right) generated predictions about incoming sensory inputs, which were projected to prediction error microcircuits (PE+ and PE-, in red and blue boxes respectively) via synaptic weights (W). These predictions were iteratively refined during inference by updating internal representations based on prediction errors and adjusting synaptic weights according to a Hebbian learning rule.(TIFF)

S3 FigSchematic for batch training of naturalistic images.A dataset of 2560 naturalistic images was partitioned into batches of 64 images. Each image within a batch was processed by an independent copy of the model (${\text{M}}_{\text{i}}$), where all copies shared a common set of synaptic weights (${\text{W}}^{\text{i}}$) during inference of the corresponding image. Following inference, the mean neural activities of excitatory cells within both prediction error and representation microcircuits were used to calculate weight updates ($\Delta W_{\text{j}}^{\text{i}}$) for each model copy (see Eq. 2). The global synaptic weight update ($\Delta {\text{W}}^{\text{i}}$) was determined by averaging these individual updates across all model copies. Subsequently, the updated weights (${\text{W}}^{\text{i}+\text{1}}$) were applied to the next batch, and the process was iterated until all batches were processed.(TIFF)

S4 Fig Oddball experiments under simulated propofol conditions. (A) The GABAergic agonistic effect of propofol is simulated by reducing the weights of L5 excitatory neuron projections to L2/3 prediction error microcircuits by 30%. Under both simulated propofol conditions (B and C), we observed reduced oscillatory power in all cortical circuits (see Fig 5B for comparison). However, a transient activity in negative prediction error microcircuit occurred (B).
(TIFF)

S1 Video Predictive coding of sensory inputs and emergent oscillations. **(Left)** Given sensory inputs (e.g., a bird), positive and negative prediction error (PE) and representation (Rep) microcircuits hierarchically interact to infer the visual object in pursuit from sensory signals (i.e., inference). **(Right)** The three lines represent mean firing rates of excitatory cells in each of the three microcircuits: L5 for Rep in Area 2 and L2/3 for PE+ and PE- from Area 1 (see Fig 1 for detailed architecture). For visual aid purpose, we show top-down predictions (gray animation) and bottom-up positive and negative PEs (red and blue animation, respectively) in the same dimension as original sensory inputs. As PE decreases, the color of animation becomes lighter and prediction becomes clearer. All three microcircuits show dampening oscillatory dynamics.
(MP4)
