# Supplementary material for: Cortical networks with multiple interneuron types generate oscillatory patterns during predictive coding
Source: PLoS Comput Biol. 2025 Sep 10;21(9):e1013469. doi: 10.1371/journal.pcbi.1013469 (PMC12443261; doi:10.1371/journal.pcbi.1013469)
Supplement: S1 Table — (DOCX) [file pcbi.1013469.s001.docx]

S1 Table. List of synaptic weights in the cortical column model of predictive coding.

| Microcircuit(s) | Source neurons | Target neurons | Plasticity | Value |
| --- | --- | --- | --- | --- |
| PE+ or PE- | L2/3 Pyr | L2/3 PV | No | 1 |
|  | L2/3 Pyr | SST |  |  |
|  | L2/3 Pyr | VIP |  |  |
|  | L2/3 PV | L2/3 Pyr |  |  |
|  | SST | L2/3 Pyr |  |  |
|  | VIP | SST |  |  |
|  | L2/3 Pyr | L2/3 Pyr |  |  |
| Rep | L4 Pyr | L4 PV | No | 1 |
|  | L4 PV | L4 Pyr |  |  |
|  | L4 Pyr | L5 Pyr |  |  |
|  | L5 Pyr | L5 Pyr |  |  |
| PE+ and Rep | L2/3 Pyr | L4 Pyr | Yes | [0, 1] |
|  | L5 Pyr | PV |  |  |
|  | L5 Pyr | SST |  |  |
|  | L5 Pyr | VIP |  |  |
| PE- and Rep | L2/3 Pyr | L4 PV | Yes | [0, 1] |
|  | L5 Pyr | L2/3 Pyr |  |  |
|  | L5 Pyr | SST |  |  |
|  | L5 Pyr | VIP |  |  |
