# Supplementary figures and images for: Cortical networks with multiple interneuron types generate oscillatory patterns during predictive coding
Source: PLoS Comput Biol. 2025 Sep 10;21(9):e1013469. doi: 10.1371/journal.pcbi.1013469 (PMC12443261; doi:10.1371/journal.pcbi.1013469)

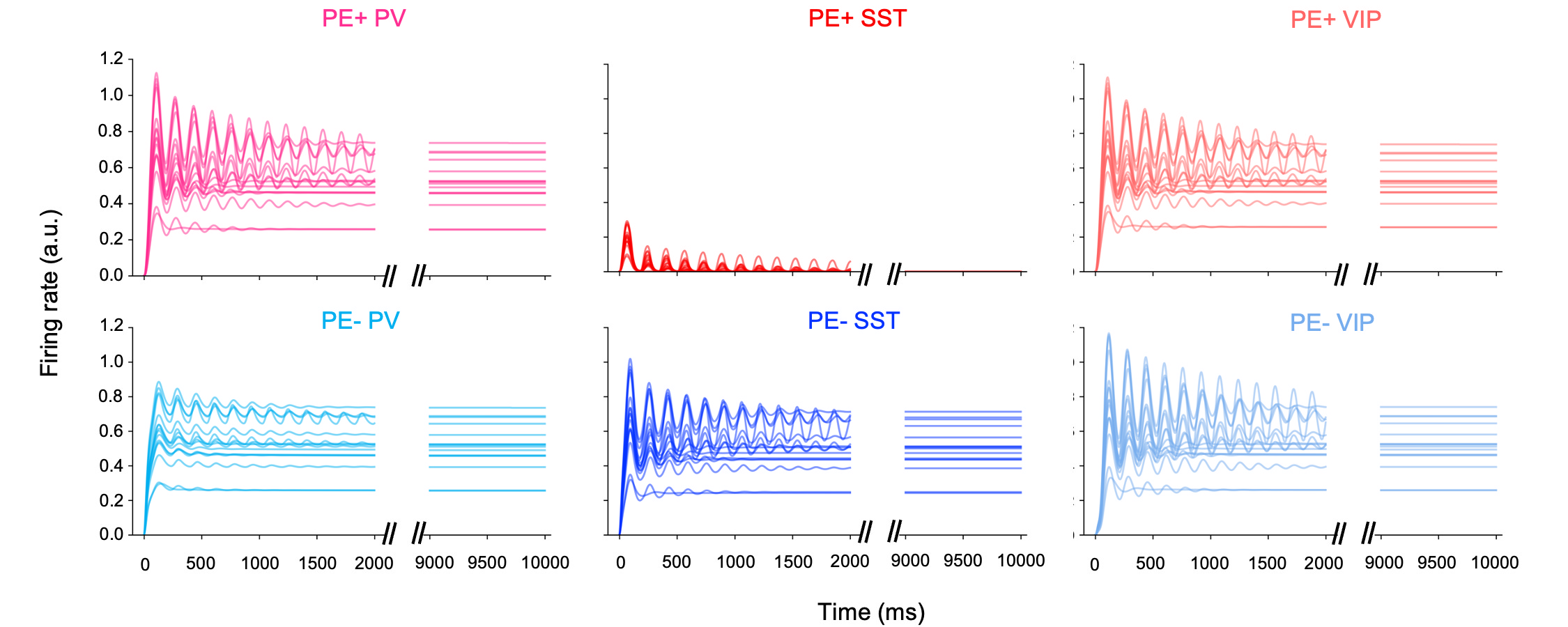

Supplement: S1 Fig — Similar to the excitatory cells (see Fig 4), each of the three interneurons (PV, SST, and VIP) in positive and negative PE microcircuits (color as in Fig 1C) also show rhythmic activities that dampen across time to reach stable focuses. (TIFF) [file pcbi.1013469.s002.tiff]

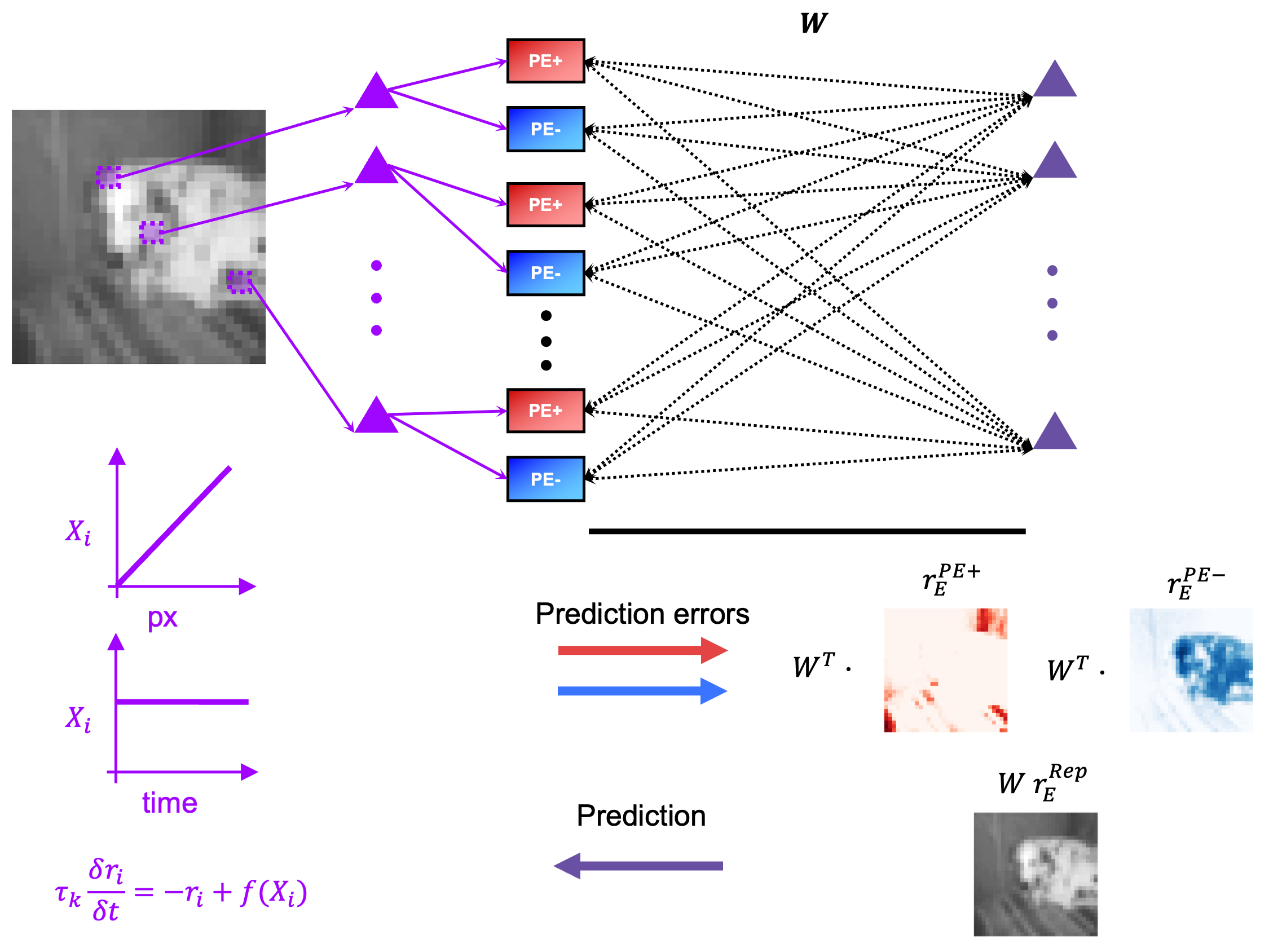

Supplement: S2 Fig — Each L4 excitatory cell in Area 1 (light purple triangles to the left) received a constant pixel-level input (Xi), representing a receptive field of one pixel. L5 excitatory cells (dark purple triangles to the right) generated predictions about incoming sensory inputs, which were projected to prediction error microcircuits (PE+ and PE-, in red and blue boxes respectively) via synaptic weights (W). These predictions were iteratively refined during inference by updating internal representations based on prediction errors and adjusting synaptic weights according to a Hebbian learning rule. (TIFF) [file pcbi.1013469.s003.tiff]

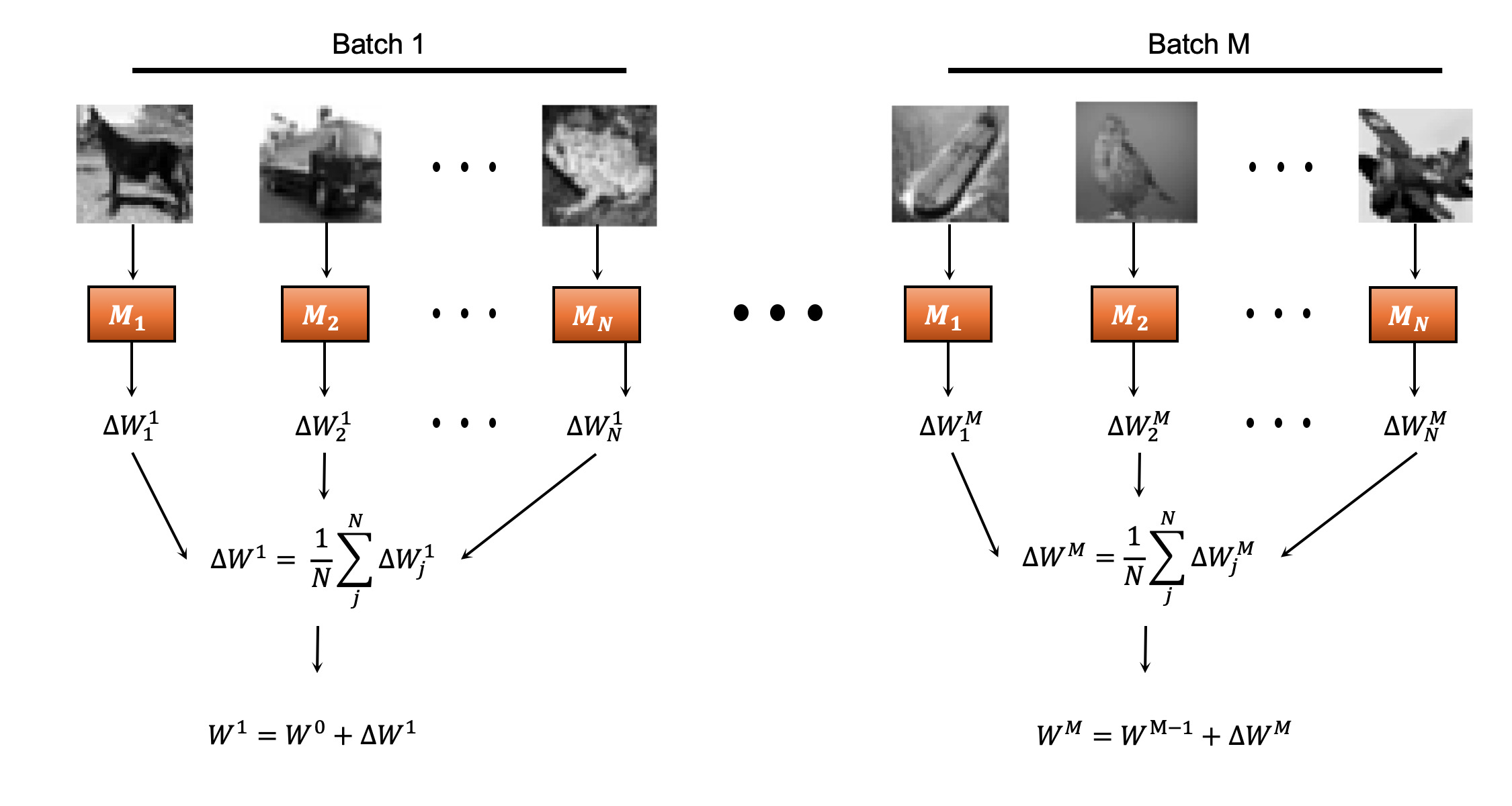

Supplement: S3 Fig — A dataset of 2560 naturalistic images was partitioned into batches of 64 images. Each image within a batch was processed by an independent copy of the model (Mi), where all copies shared a common set of synaptic weights (Wi) during inference of the corresponding image. Following inference, the mean neural activities of excitatory cells within both prediction error and representation microcircuits were used to calculate weight updates (ΔWji) for each model copy (see Eq. 2). The global synaptic weight update (ΔWi) was determined by averaging these individual updates across all model copies. Subsequently, the updated weights (Wi+1) were applied to the next batch, and the process was iterated until all batches were processed. (TIFF) [file pcbi.1013469.s004.tiff]

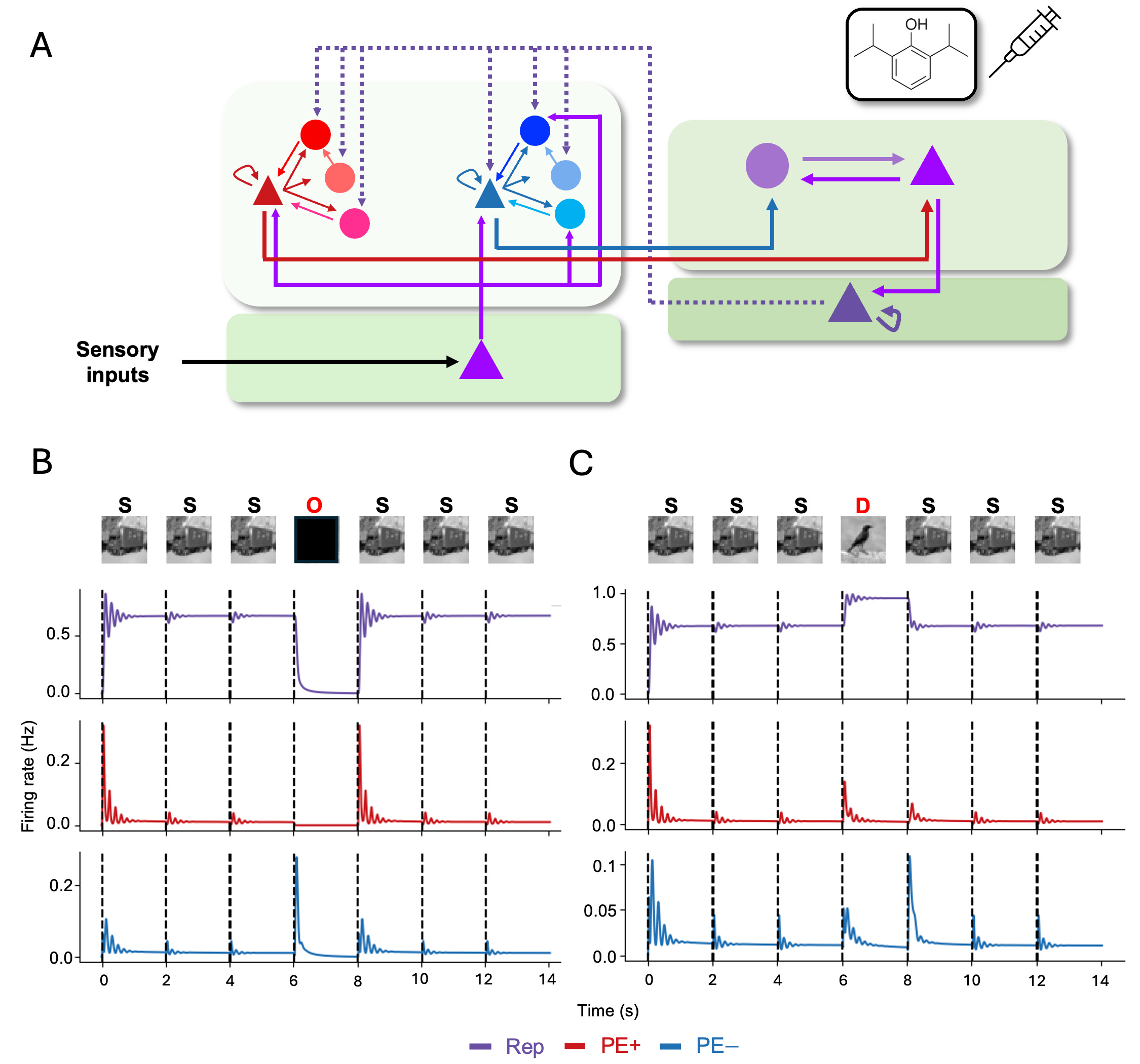

Supplement: S4 Fig — (A) The GABAergic agonistic effect of propofol is simulated by reducing the weights of L5 excitatory neuron projections to L2/3 prediction error microcircuits by 30%. Under both simulated propofol conditions (B and C), we observed reduced oscillatory power in all cortical circuits (see Fig 5B for comparison). However, a transient activity in negative prediction error microcircuit occurred (B). (TIFF) [file pcbi.1013469.s005.tiff]
